# Using Health-Related Social Media to Understand the Experiences of Adults With Lung Cancer in the Era of Immuno-Oncology and Targeted Therapies: Observational Study

**DOI:** 10.2196/45707

**Published:** 2023-07-12

**Authors:** Alison Booth, Stephanie Manson, Sonia Halhol, Evie Merinopoulou, Mireia Raluy-Callado, Asha Hareendran, Stefanie Knoll

**Affiliations:** 1 Data Analytics Evidera London United Kingdom; 2 Health Economics, Outcomes Research (HEOR), Novartis Oncology East Hanover, NJ United States

**Keywords:** non-small cell lung cancer, data science, machine learning, natural language processing, social media data, patient experience, patient preference, immunotherapy, targeted therapies, lung cancer, social media

## Abstract

**Background:**

The treatment of non–small cell lung cancer (NSCLC) has evolved dramatically with the approval of immuno-oncology (IO) and targeted therapies (TTs). Insights on the patient experience with these therapies and their impacts are lacking. Health-related social media has been increasingly used by patients to share their disease and treatment experiences, thus representing a valuable source of real-world data to understand the patient’s voice and uncover potential unmet needs.

**Objective:**

This study aimed to describe the experiences of patients with NSCLC as reported in discussions posted on lung cancer–specific social media with respect to their disease symptoms and associated impacts.

**Methods:**

Publicly available posts (2010-2019) were extracted from selected lung cancer– or NSCLC-specific websites. Social media users (patients and caregivers posting on these websites) were stratified by metastatic- and adjuvant-eligible subgroups and treatment received using natural language processing (NLP) and machine learning methods. Automated identification of symptoms was conducted using NLP. Qualitative data analysis (QDA) was conducted on random samples of posts mentioning pain-related, fatigue-related, respiratory-related, or infection-related symptoms to capture the patient experience with these and associated impacts.

**Results:**

Overall, 1724 users (50,390 posts) and 574 users (4531 posts) were included in the metastatic group and adjuvant group, respectively. Among users in the metastatic group, pain, discomfort, and fatigue were the most commonly mentioned symptoms (49.7% and 39.6%, respectively), and in the QDA (258 posts from 134 users), the most frequent impacts related to physical impairments, sleep, and eating habits. Among users in the adjuvant group, pain, discomfort, and respiratory symptoms were the most commonly mentioned (44.8% and 23.9%, respectively), and impacts identified in the QDA (154 posts from 92 users) were mostly related to physical functioning.

**Conclusions:**

Findings from this exploratory observational analysis of social media among patients and caregivers informed the lived experience of NSCLC in the era of novel therapies, shedding light on most reported symptoms and their impacts. These findings can be used to inform future research on NSCLC treatment development and patient management.

## Introduction

### Background

Lung cancer is the leading cause of cancer mortality worldwide and is second in cancer incidence, with an estimated 1.8 million deaths (18.0% of total cancer deaths) and 2.2 million new cases (11.4% of total cancer cases) in 2020 [1]. In the United States, lung cancer is also the second most commonly diagnosed cancer and the leading cause of cancer deaths, and the National Cancer Institute estimates 235,760 incident cases in 2021 [2]. There are 2 distinct histopathological types of lung cancer: small cell lung cancer and non–small cell lung cancer (NSCLC); the latter accounts for approximately 84% of lung cancer cases [3].

### Treatment Landscape

Treatment for NSCLC varies by stage of the disease. In general, patients with early-stage resectable NSCLC undergo surgery with or without (+/-) adjuvant therapy, while patients with advanced or metastatic NSCLC have been traditionally treated with chemotherapy [4]. However, treatment approaches have drastically shifted over the past decade, notably with the emergence of several molecular-targeted and immuno-oncology (IO) agents. Despite the progress of these improved treatments, the prognosis of NSCLC remains poor [5].

### Prior Works

Previous studies that assessed patient-reported symptom burden and impacts on health-related quality of life among NSCLC populations using lung cancer–specific and generic scales have highlighted a significant unmet need with the current treatment options [6,7].

### Rationale

Health-related social media (ie, lung cancer-specific forums) present a rich source of real-world evidence from the individual perspective that can inform research aiming to understand the overall patient journey through their disease, including but not limited to symptom burden, real-world treatment use, impact on quality of life, and other important issues and concerns. Social media, specifically health-related social media, has become an increasingly common resource used by patients and caregivers to share their journeys and experiences. In June 2018, the United States Food and Drug Administration published a draft guidance encouraging stakeholders to explore the use of social media when conducting studies, particularly to shed light on patients’ perspectives and experiences [8,9]. Furthermore, a study comparing 4 methods for obtaining patient-reported outcomes (PROs) to capture patient experiences, including social media, found social media to uncover the most concepts and be the least resource-intensive of the 4 methods.

### Goal of This Study

Using publicly available discussions in lung cancer-specific social media, this study aimed to better understand the experience of patients with NSCLC in adjuvant and advanced or metastatic (stage IIIb/IV) stages with regard to their symptoms and symptom impacts.

## Methods

### Overview

This was an exploratory retrospective analysis of existing publicly available discussions posted between January 2010 and November 2019 on health-related social media websites among patients with self-reported adjuvant or advanced or metastatic NSCLC or their caregivers. In this study, users of the websites were patients and their caregivers (eg, parents, children, and siblings). The decade from 2010 was chosen to reflect the period of the majority of approvals of IO and targeted therapies (TTs) for NSCLC by the Food and Drug Administration.

### Selection Criteria

#### Inclusion Criteria

Social media users (self-identified as patients or caregivers) were included in the study if they started posting on the following lung cancer– or NSCLC-specific social media websites (subforums) between January 2010 and November 2019: MacMillan Cancer Support (lung cancer), LUNGevity Lung Cancer Support Community (NSCLC), Health Boards (lung cancer), Cancer Survivors Network (lung cancer), and Cancer Compass (lung cancer).

All lung cancer–specific social media hosted in the United States or the United Kingdom were initially screened. Generic social media websites (eg, Facebook, Google+, and Twitter) were not considered because of the added complication of filtering out irrelevant material, as were those that used languages other than English.

#### Exclusion Criteria

Social media users were excluded if they mentioned small cell lung cancer in their posting history or began posting on the website before 2010.

### Data Management

Posts in the public domain on the included social media websites were programmatically extracted using validated algorithms in the R Statistical Programming Language (R Core Team). Upon extraction, data were deidentified by removal of identifiable personal information (name, postcode or ZIP, place names, email addresses, phone numbers, or social security numbers) and conversion of raw usernames to unique identifiers. Data were also processed to correct for misspellings, remove non–UTF-8 text, remove duplicate posts, and standardize all drug names to generic names.

### Study Subgroups

Social media users who fulfilled the inclusion and exclusion criteria were assigned to a stage-specific subgroup, and within these, they were further classified by the treatment class received: (1) adjuvant, in which patients had had surgery and were subsequently treated with chemotherapy, IO, TT, or radiation therapy (RTx), and (2) advanced or metastatic, in which patients were treated with chemotherapy, IO, or TT. Tables 1 and 2 provide the definition of each treatment-specific subgroup by adjuvant and metastatic stage, respectively. Drugs within each treatment class by stage are listed in Tables S1 and S2 in Multimedia Appendix 1.

Stage-specific subgroups (adjuvant and metastatic) were mutually exclusive, while treatment class subgroups were not, since patients could report their experience with more than one treatment class within the study period, with the exception of the surgery +/- RTx-only subgroup that included those users who did not mention any chemotherapy, IO, or TT drug.

**Table 1:** Adjuvant subgroups definitions.

Subgroup			Definition
**Adjuvant NSCLC^a^ subgroup**			Mention of adjuvant- or surgery-related terms and no mention of stage IIIb/IV or metastatic terms
	Treated with surgery +/- RTx^b^ only	No mention of treatment following surgery	
	Treated with chemotherapy	Mention of a chemotherapy drug indicated at the adjuvant setting following surgery or mention of unspecified “chemotherapy,” and no mention of an IO^c^ or TT^d^ indicated at the adjuvant setting or in clinical trials following surgery	
	Treated with IO or TT	Mention of an IO or TT indicated at the adjuvant setting or in clinical trials following surgery	

^a^NSCLC: non-small cell lung cancer.

^b^RTx: radiation therapy.

^c^IO: immuno-oncology.

^d^TT: targeted therapy.

**Table 2:** Metastatic subgroups definitions.

Subgroup			Definition
**Metastatic NSCLC^a^ subgroup**			Mention of stage IIIb/IV or a metastatic term and a treatment indicated at the metastatic setting
	Treated with IO^b^	Mention of a corresponding IO drug	
	Treated with TT^c^	Mention of a corresponding TT drug	
	Treated with chemotherapy	Mention of a corresponding chemotherapy drug	

^a^NSCLC: non-small cell lung cancer.

^b^IO: immuno-oncology.

^c^TT: targeted therapy.

### Data Analysis

#### Subgroup Identification

The identification of the study subgroups was driven by the data. Indeed, terms were used to subset users into their corresponding subgroups using natural language processing (NLP). Only social media posts in which social media users mentioned receiving applicable treatments or surgeries were selected. NLP algorithms were developed using the WordVectors [10] R packages to generate clusters of similar words to aid in the identification of relevant stage- and surgery-related terms (eg, “stage III,” “stage IV” or “advanced stage,” and “lobectomy”) within the data. In addition, frequencies of n-grams (unigrams, bigrams, and trigrams) within the data were generated to aid in the identification of multiword terms used to describe relevant terms.

#### Machine Learning Analyses

In order to ensure that users were referring to true treatment experiences when mentioning symptoms, machine learning (ML) techniques were applied to predict whether sentences that mention a treatment of interest were referring to an actual treatment experience. The input consisted of individual sentences, as opposed to entire posts, which are quite often lengthy and involve a mix of true and untrue experiences. By using sentences, we ensured that only the true treatment experience statements were used for training the ML algorithms. Posts that were predicted to not relate to actual NSCLC treatment experiences (except for the surgery +/- RTx-only adjuvant subgroup) were removed.

#### Automated Symptom Identification

Automated symptom identification was conducted for all included social media users by subgroup. Posts included in the symptom identification were required to have at least one mention of any one of the treatments of interest (Tables S1 and S2 in Multimedia Appendix 1). Symptoms were captured using the Apache clinical Text Analysis Knowledge Extraction System, an NLP tool mapping concepts from the Uniform Medical Language System to clinical terms in posts, developed by the Apache Software Foundation. Custom lexicons were used to supplement clinical Text Analysis Knowledge Extraction System to capture lay terms present in social media data. The proportions of patients experiencing a symptom were calculated as the number of social media users who mentioned a symptom and a specific treatment out of all social media users who mentioned the respective treatment.

#### Qualitative Data Analyses

Qualitative data analysis (QDA) was conducted on samples of social media users posting histories for each subgroup. For specificity to the objectives, only posts containing a mention of one or more pain-related (metastatic subgroup only), fatigue-related, respiratory-related, or infection-related symptoms were sampled for the QDA. QDA was conducted in ATLAS.ti (version 8.4.4), developed by Thomas Muhr and Scientific Software Development GmbH. Thematic analysis principles were followed [11,12]. Codes were reviewed, synthesized, and assigned to data-driven themes, categories, and subcategories. Samples of social media users were randomly generated, and their posts were analyzed until saturation was reached.

### Ethical Considerations

To date, no firm guidelines on the use of health-related social media data exist; however, this study followed available published ethics frameworks [13,14]. Only publicly accessible sources were used (ie, no login was required to access the material), and the terms and conditions were reviewed to ensure compliance. Following the University of Sheffield Ethics guidelines on the identification of subjects observed in the public setting (Research Ethics Policy Note on Principles of Consent) [14], all measures were taken to ensure anonymity and that no user-generated content was reproduced verbatim.

### Data Confidentiality

Because of the nature of the data used for this study, patient consent and ethical approval were not required. All data collected in the study were kept strictly confidential and were fully anonymized (see the *Data Management* section for details). The data collected included usernames (programmatically replaced with unique anonymous IDs), message content, URLs of posts used for quality assurance, and posting dates. Any other metadata on how users interact with the website, such as location data, IP addresses, and so on, were neither collected nor stored. Personal data were removed programmatically, and no posting content was reproduced verbatim in any dissemination; all quotes used were paraphrased to ensure confidentiality. Finally, no researchers registered with any of the sources to gain access to the data, nor did any researchers post to the data sources.

## Results

### Study Population

A total of 14,060 social media users (153,991 posts) were identified. After applying all selection criteria, ML, and NLP, 2298 social media users (54,921 posts) remained and were assigned to adjuvant (574 users; 4531 posts) and metastatic (1724 users; 50,390 posts) subgroups (Table 3).

**Table 3:** Study population.

Subgroup		Users, n	Posts, n
**Adjuvant NSCLC^a^ subgroup^b^**		574	4,531
	Treated with surgery +/- RTx^a,c^ only	289	755
	Treated with chemotherapy	282	3754
	Treated with IO^d^ or TT^e^	27	579
**Metastatic NSCLC subgroup^b^**		1724	50,390
	Treated with IO	170	16,570
	Treated with TT	423	26,475
	Treated with chemotherapy	1589	49,616

^a^NSCLC: non-small cell lung cancer.

^b^Staging subgroups are mutually exclusive, treatment groups within those are not, with the exception of the surgery +/- RTx only subgroup.

^c^RTx: radiation therapy.

^d^IO: immuno-oncology.

^e^TT: targeted therapy.

### Adjuvant Subgroup

#### Automated Identification of Symptoms

**Figure 1:**
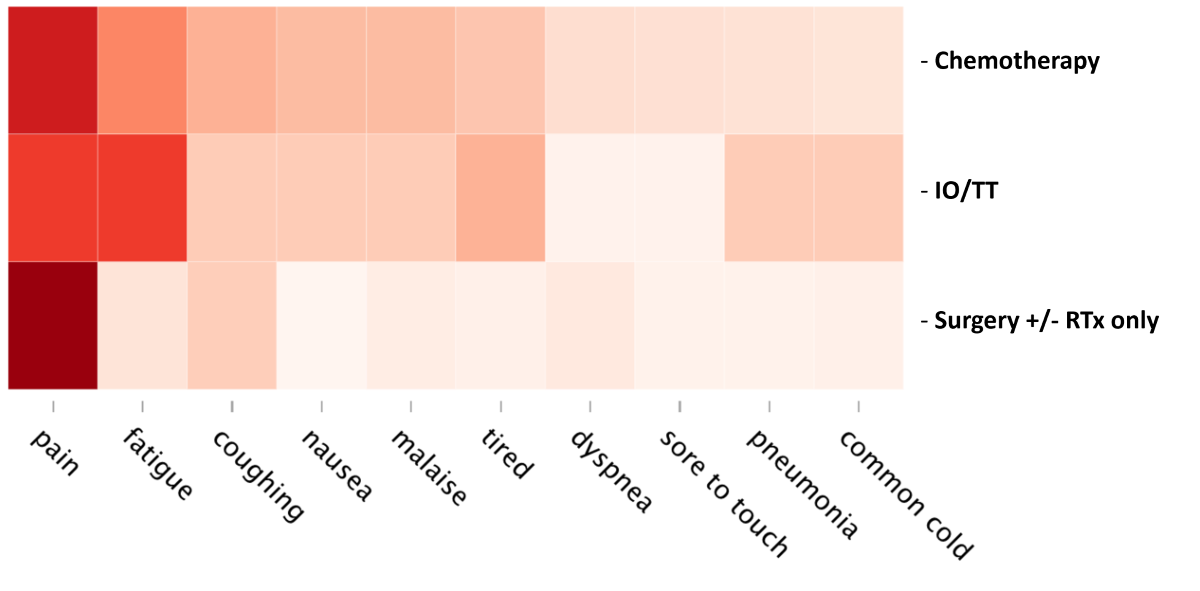
The 10 most frequently mentioned symptoms by patients with adjuvant non-small cell lung cancer (NSCLC) or their caregivers using publicly available health-related social media, by treatment group. IO: immuno-oncology; RTx: radiation therapy; TT: targeted therapy.

The summary results of the automated symptom extraction can be found in Figure 1. Among adjuvant chemotherapy social media users, the most frequently discussed symptoms were pain (34.4%), fatigue (20.6%), and coughing (14.9%). Among adjuvant IO or TT social media users, the most frequently discussed symptoms were pain (29.6%), fatigue and tiredness (29.6% and 14.8%, respectively), and pleural diseases (14.8%). Among surgery +/- RT-only social media users, the most frequently mentioned symptoms were pain (40.2%), coughing (10.9%), and fatigue (7.1%).

#### QDA Outcomes

A total of 92 adjuvant social media users (154 posts) were included in the QDA (surgery ± RTx only=41 users [62 posts]; chemotherapy=43 users [75 posts]; and IO or TT=8 users [17 posts]), at which point saturation was considered to have been reached. Categories identified in the analyses for chemotherapy and IO or TT social media users were physical impacts, emotional impairments, impacts on eating, impacts on sleep, impacts on health and well-being, and impacts on work. Commonly mentioned symptom impacts among patients who received adjuvant treatment were the need to sleep more than usual due to fatigue, having difficulty walking and standing due to pain or weakness, being unable to eat due to loss of appetite, and feeling frustrated due to symptoms such as fatigue (Table 4). For surgery ± RTx social media users, only physical impairments, impacts on sleep, and impacts on eating were identified.

**Table 4:** Symptom-related impacts identified in the qualitative analyses of a sample of patients who received adjuvant treatment for non–small cell lung cancer (NSCLC) or their caregivers using publicly available health-related social media (N=51 users).

Category	Physical impairments	Emotional impacts	Impacts on eating	Impacts on sleep	Impacts on health and well-being	Impacts on work
Finding^a^	Users reported difficulty walking or standing due to pain or weakness. Weight loss as a result of appetite changes was also reported.	Users reported feeling frustrated, depressed, and sometimes powerless about their symptoms.	Users reported not being able to eat or having little or no appetite.	Some users reported being so tired they needed to sleep all the time, whereas others reported struggling to sleep due to pain.	Users reported struggling in general or not feeling well as a result of their symptoms.	Users reported finding work difficult or their performance negatively impacted as a result of their symptoms.
Example^b^	“He is suffering with severe ankle pain, and it makes it very difficult for him to walk” [caregiver] “I do not have much appetite. I’ve lost around 5 kilograms” [patient]	”I find the hair loss depresses me...” [patient] “The epileptic fits leave me feeling powerless for a while” [patient with metastases to brain]	“Food he used to like he now turns his nose up at. He’s only able to eat one bite and then does not eat any more” [caregiver]	“I’ve been sleeping nearly all day every day. I feel way too tired to do anything” [patient] “The pain is terrible so I am having to try to sleep differently as I struggle on my side” [patient]	“I am really struggling with the exhaustion [patient] “Her lung infections have been there for a while, which most likely explains why she felt so terrible” [patient]	“I did not have a good day at work today as I feel sick and tired all the time” [patient]

^a^These symptom impacts were observed among patients with NSCLC and their caregivers who are using publicly available health-related social media and may not be representative of the whole NSCLC population.

^b^Quotations are paraphrased to protect users’ privacy.

### Metastatic Subgroup

#### Automated Identification of Symptoms

**Figure 2:**
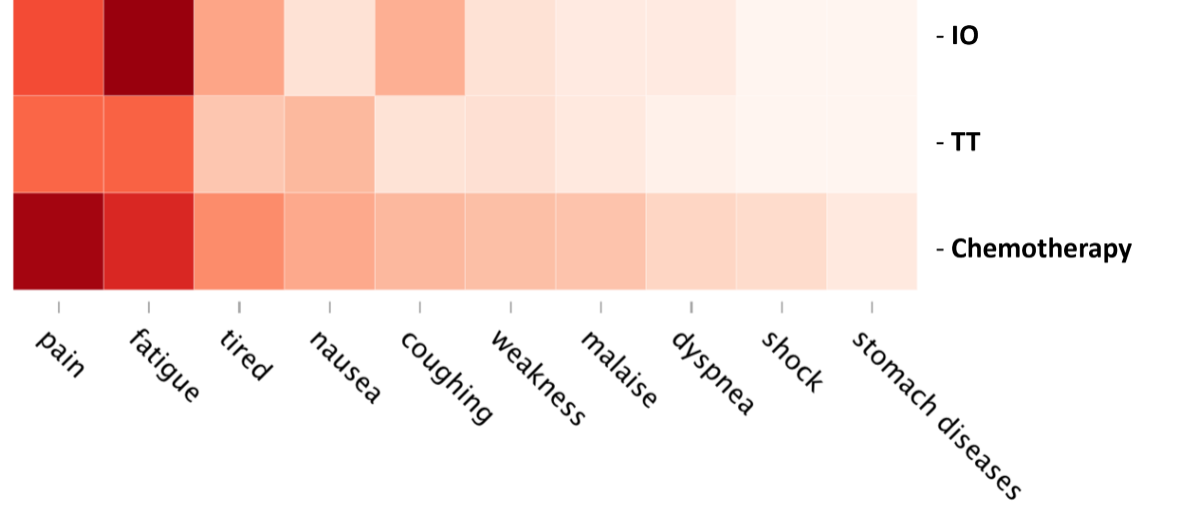
The 10 most frequently mentioned symptoms by patients with metastatic non-small cell lung cancer (NSCLC) or their caregivers using publicly available health-related social media, by treatment group. IO: immuno-oncology; TT: targeted therapy.

The summary results of the automated symptom extraction can be found in Figure 2. Among the chemotherapy subgroup, the most frequently discussed symptoms were pain (38.4%), fatigue and tiredness (33.0% and 22.0%, respectively), and nausea (18.4%). Among the IO subgroup, the most frequently discussed symptoms were fatigue (39.4%), pain (28.8%), and coughing (17.7%). Among the TT subgroup, the most frequently mentioned symptoms were fatigue (26.5%), pain (26.0%), and diarrhea (17.7%). The proportion of social media users in the metastatic subgroup mentioning the 25 most common symptoms can be found in Table S4 in Multimedia Appendix 1.

#### QDA Outcomes

A total of 134 metastatic users (258 posts) were included in the QDA (chemotherapy=42 users [91 posts]; IO=42 users [87 posts]; IO or TT=50 users [80 posts]), at which point saturation was considered to have been reached. Identified categories of symptom impacts included physical impairments, impacts on sleep, eating, day-to-day activities, and emotional impacts (Table 5). The most frequently reported symptom impacts across all subgroups were the need to rest and sleep a lot due to fatigue or weakness, waking up at night, and not having enough energy to eat.

**Table 5:** Symptom-related impacts identified in the qualitative analyses of a sample of patients with metastatic non–small cell lung cancer (NSCLC) or their caregivers using publicly available health-related social media (N=134 users).

Category	Physical impairments	Impacts on sleep	Impacts on eating	Impact on family	Impact on activities	Emotional impacts
Finding^a^	Users reported difficulty performing day-to-day tasks, having mobility issues, and being unable to get out of bed due to weakness and fatigue.	Some users report being so tired they sleep all the time, whereas others report their symptoms such as pain and cramp prevent them from sleeping.	Users reported a reduction in appetite. For some users this was associated with a bad taste in the mouth, and others due to lack of energy or sleeping all the time.	Impacts on families were reported by patients and caregivers. These ranged from increased burden on family to changes in family dynamics.	Practical impacts reported included changes in normal life, ranging from needing to change skin or hygiene products to not being able to go out.	Patients expressed that the symptoms they experience have subsequent impacts on their emotions and mental health.
Example^b^	“He was so weak that he could not even sit up in his bed without help” [caregiver] “I’ve been in bed for the last two days, feeling nauseous and retching, with extreme lethargy” [patient] “She becomes quite out of breath and tired so she needs to rest a lot” [caregiver]	“I have major cramps, it makes it harder to sleep at night” [patient] “For years I have had chronic pain and struggle with sleep. I have medication but it doesn’t always help” [patient] “the fatigue is my worse symptom, I sleep into the afternoon all the time” [patient]	“I did not like eating because I just was too tired” [patient] “I am struggling to eat as the metallic taste in my mouth makes a lot of the food taste horrible” [patient] “he finds it hard to stay awake to eat so we are giving him high energy foods when he can eat” [caregiver]	“worried how I will cope at home…I hate this disease” [caregiver] “I miss playing with my kid.. He understands that daddy needs to rest but it breaks my heart” [patient] “His personality change made me start questioning my marriage.” [caregiver]	“he hasn’t been able to do much for ages. It means we don’t really go out or see people” [caregiver] “the skin reaction is really bad and I have to be really careful what products I use” [patient] “the sweating is so bad he has to change clothes multiple times per day” [caregiver]	“Suffers with depression and anxiety as he is unable to do anything. Prior to this, he was busy and active” [caregiver] “The on and off hair loss is upsetting me” [patient] “he doesn’t want visitors...he doesn’t want to pretend to be in good spirits” [caregiver]

^a^These symptom impacts were observed among patients with NSCLC and their caregivers who are using publicly available health-related social media and may not be representative of the whole NSCLC population.

^b^Quotations are paraphrased to protect users’ privacy.

## Discussion

### Principal Results

In this exploratory NLP and QDA of posts extracted from publicly available health-related social media by patients with NSCLC in the adjuvant setting and their caregivers, the most mentioned symptom was pain, irrespective of treatment status (surgery +/- RTx only or receiving adjuvant treatment). Among users of social media who received surgery +/- RTx only, mentions of respiratory-related symptoms (such as cough and pneumonia) appeared more common than among patients who received adjuvant NSCLC treatment. Symptoms, including pain and fatigue, appeared to be more commonly mentioned among patients who received adjuvant NSCLC treatment than among users who received surgery +/- RTx only. The QDA identified that symptoms were often associated with negative impacts, such as inability to exercise, difficulty sleeping, and taking time off work among surgery +/- RTx-only social media users and difficulty walking, feeling frustrated, oversleeping, and having difficulty at work among the adjuvant subgroup who received treatment after surgery.

In the analysis of posts by patients with metastatic NSCLC or their caregivers, the pain was likewise mentioned as the most common symptom, followed by fatigue, irrespective of the treatment group. The QDA identified that pain and fatigue were frequently mentioned in relation to increased difficulty in performing day-to-day tasks or getting out of bed, reduced interactions with family, and impacting patients’ ability to eat. Furthermore, social media users reported an increased burden on family members due to these symptoms, as patients often required assistance in performing routine tasks.

The analysis contains patients’ and caregivers’ first-hand experiences, which are described in a setting with no researcher or medical professional present. Results are therefore likely to reflect the true opinions of social media users, as the data are less likely to be impacted by information bias. It is also likely that the topics most mentioned by patients and caregivers using publicly available health-related social media represent those that are of the greatest importance to them and have the biggest impact on their lives since the topics discussed are driven by patients and caregivers. A deeper understanding of the patient experience may lead to positive impacts on patient and physician discussions.

### Limitations

Limitations to this exploratory study, including potential selection biases in relation to the user profiles of those posting on health-related social media, are not well understood, and there may be some bias in the information patients share in the public domain. It should be noted that insights derived from this study represent the population of patients with NSCLC or their caregivers who are using publicly available health-related social media and may not be representative of the whole NSCLC population and are therefore not generalizable. However, the nature of this study was hypothesis-generating and exploratory. While the study described experiences based on certain treatments of interest, the sample sizes in each treatment group were too small to draw comparisons, and no statistical tests were conducted to assess differences between treatment groups. Moreover, treatment groups were not mutually exclusive, and it is possible that there could be some misclassification in grouping users. Results by the treatment group should be interpreted with caution and should be used as hypothesis-generating qualitative insights. Furthermore, users posting on more than one included website were handled to the extent possible (using duplicate postings and usernames); however, there is the potential that some duplicate users remained in the analyses. Symptom rates should not be used as proxy calculations for symptom incidence, as these are limited to reports from health-related social media. While there are acknowledged limitations to the use of social media data, a study comparing the method to qualitative interviews and group concept mapping concluded that each method has stand-alone merit for specific research questions and that the use of multiple methods combined resulted in a deeper understanding of the patient experience.

### Comparison With Prior Work

Fatigue and pain have been historically reported as the most common symptoms and side effects among patients with NSCLC at all stages, and effective treatment remains a challenge [15-17]. Our study suggests that pain and fatigue are not only the most common symptoms or side effects but also among the most bothersome to patients, as measured by social media posting activity. This underlines the importance of understanding how treatments are likely to impact pain and fatigue. Other recent studies have focused on the clinical outcomes of patients with advanced or metastatic disease treated with IO or TT [18]. A 2018 study assessed patient-reported symptoms and treatment impacts through administered PRO tools and questionnaires among patients with advanced NSCLC treated with chemotherapy or TT (osimertinib) in the AURA3 phase III trial. The study investigated improvements in prespecified symptoms and reported that almost 60% of patients on osimertinib noted an improvement in fatigue compared to approximately 40% of patients on chemotherapy (odds ratio 1.96, *P*=.008) [19].

### Conclusions

This study used information from publicly available lung cancer-related social media to gain insights into the experiences of patients with NSCLC and their caregivers. Some of the key insights gained in this exploratory study were the important burden of pain and fatigue on patients across treatment groups, the high frequency of respiratory symptoms, and the impact of those symptoms on patients’ daily functioning. Such insights shed light on the unmet needs of patients and their caregivers, allowing researchers to better understand the challenges they face in relation to the management of disease symptoms and their decision-making about treatment options.

Using this information, researchers can begin to address those needs that are of the greatest importance to patients and their caregivers and ensure data are collected on these concepts in the tools used to evaluate patient outcomes and experiences. Findings from health-related social media could be considered in the selection of PRO measures or domains to include by identifying symptoms that are prioritized by patients for discussion in the era of TT and IO. The findings of this study could be explored further and validated in future research and also help to understand the patient’s needs for consideration in future NSCLC development programs.

## Appendix

Multimedia Appendix 1 Treatments of Interest and Symptoms Rates.

## Abbreviations

IO: immuno-oncology
ML: machine learning
NLP: natural language processing
NSCLC: non–small cell lung cancer
QDA: qualitative data analysis
PRO: patient-reported outcome
RTx: radiation therapy
TT: targeted therapy

## Data Availability

Due to concerns about being able to use web-based search engines to trace the original social media user based on the post text in the raw data sets, the authors will not be making the data set available.
