# Supplementary material for: Using Health-Related Social Media to Understand the Experiences of Adults With Lung Cancer in the Era of Immuno-Oncology and Targeted Therapies: Observational Study
Source: JMIR Cancer. 2023 Jul 12;9:e45707. doi: 10.2196/45707 (PMC10372558; doi:10.2196/45707)
Supplement: Multimedia Appendix 1 [file cancer_v9i1e45707_app1.docx]

Multimedia Appendix 1

Table S1. Adjuvant Treatments of Interest for NSCLC.

| **Treatment Type** | **Treatment Class** | **Drug** |
| --- | --- | --- |
| Monotherapy | Chemotherapy | Vinorelbine |
|  | TT | Erlotinib^a^  Gefitinib^a^  MAGE-A3^a^ |
|  | IO | Pembrolizumab^a^  Durvalumab^a^  Atezolizumab^a^  Nivolumab^a^ |
| Combinations | Chemotherapy | Cisplatin/vinorelbine  Cisplatin/etoposide  Cisplatin/gemcitabine  Cisplatin/docetaxel  Cisplatin/pemetrexed  Carboplatin/paclitaxel  Carboplatin/gemcitabine  Carboplatin/pemetrexed  Cisplatin/vinorelbine |
|  | TT | Bevacizumab/cisplatin/(vinorelbine or docetaxel or gemcitabine or pemetrexed) ^a^  Chemotherapy/cetuximab |

Abbreviations: IO = immuno-oncology; NSCLC = non-small cell lung cancer; TT = targeted therapy

^a^ Treatments only available in the clinical trial setting at the time of conducting the analyses.

Table S2. Treatments of Interest for Advanced/Metastatic NSCLC.

| **Treatment Type** | **Treatment Class** | **Drug** |
| --- | --- | --- |
| Monotherapy | Chemotherapy | Docetaxel  Pemetrexed  Paclitaxel  Vinorelbine tartrate |
|  | TT | Afatinib  Alectinib  Brigatinib  Ceritinib  Crizotinib  Dabrafenib  Dacomitinib  Erlotinib  Everolimus  Gefitinib  Lorlatinib  Osimertinib  Osimertinib mesylate  Ramucirumab |
|  | IO | Atezolizumab  Durvalumab  Nivolumab  Pembrolizumab |
| Combinations | Chemotherapy | Carboplatin/gemcitabine  Carboplatin/nab albumin-bound paclitaxel  Carboplatin/docetaxel  Carboplatin/paclitaxel  Carboplatin/pemetrexed  Cisplatin/docetaxel  Cisplatin/paclitaxel  Cisplatin/gemcitabine  Cisplatin/pemetrexed  Cisplatin/vinorelbine  Methotrexate/mechlorethamine/procarbazine  Necitumumab/cisplatin  Necitumumab/gemcitabine |
|  | TT | Dabrafenib/trametinib  Afatinib/cisplatin  Afatinib/carboplatin  Bevacizumab/paclitaxel/cisplatin  Ramucirumab/docetaxel |
|  | IO | Nivolumab/ipilimumab  Pembrolizumab/pemetrexed  Pembrolizumab/carboplatin/paclitaxel  Atezolizumab bevacizumab  Atezolizumab/bevacizumab/carboplatin/paclitaxel |

Abbreviations: IO = immuno-oncology; NSCLC = non-small cell lung cancer; TT = targeted therapy

## Symptom Rates

Table S3. 25 Most Commonly Mentioned Symptoms among users in the Adjuvant Cohort by Treatment Group.

|  | **Chemotherapy** | | **IO/TT** | | **Surgery +/- RTx Only** | |
| --- | --- | --- | --- | --- | --- | --- |
|  | **n** | **%** | **n** | **%** | **n** | **%** |
| Pain | 97 | 34.40% | 8 | 29.60% | 96 | 40.20% |
| Fatigue | 58 | 20.60% | 8 | 29.60% | 17 | 7.10% |
| Coughing | 42 | 14.90% | 3 | 11.10% | 26 | 10.90% |
| Nausea | 38 | 13.50% | 3 | 11.10% | 7 | 2.90% |
| Malaise | 38 | 13.50% | 3 | 11.10% | 12 | 5.00% |
| Tired | 34 | 12.10% | 4 | 14.80% | 10 | 4.20% |
| Dyspnea | 24 | 8.50% | 1 | 3.70% | 14 | 5.90% |
| Sore to touch | 23 | 8.20% | 1 | 3.70% | 9 | 3.80% |
| Pneumonia | 22 | 7.80% | 3 | 11.10% | 9 | 3.80% |
| Common cold | 20 | 7.10% | 3 | 11.10% | 10 | 4.20% |
| Stomach diseases | 20 | 7.10% | 1 | 3.70% | 14 | 5.90% |
| Spots on skin | 19 | 6.70% | 2 | 7.40% | 2 | 0.80% |
| Weakness | 17 | 6.00% | 1 | 3.70% | 7 | 2.90% |
| Swelling | 17 | 6.00% | 3 | 11.10% | 7 | 2.90% |
| Actual discomfort | 16 | 5.70% | 0 | 0.00% | 9 | 3.80% |
| Seizures | 15 | 5.30% | 1 | 3.70% | 7 | 2.90% |
| Shock | 15 | 5.30% | 0 | 0.00% | 12 | 5.00% |
| Fear | 14 | 5.00% | 2 | 7.40% | 10 | 4.20% |
| Stress | 9 | 3.20% | 3 | 11.10% | 11 | 4.60% |

Abbreviations: IO = immuno-oncology; RTx = radiation therapy; TT = targeted therapy

Please note that these symptoms were observed among patients with NSCLC and their caregivers who are using publicly available health-related social media and may not be representative of the whole NSCLC population.

Table S4. 25 Most Commonly Mentioned Symptoms Among Users in the Metastatic Cohort by Treatment Group.

|  | **Immuno-oncology** | | **Targeted therapy** | | **Chemotherapy** | |
| --- | --- | --- | --- | --- | --- | --- |
|  | **n** | **%** | **n** | **%** | **n** | **%** |
| Pain | 49 | 28.80% | 110 | 26.00% | 610 | 38.40% |
| Fatigue | 67 | 39.40% | 112 | 26.50% | 525 | 33.00% |
| Tired | 32 | 18.80% | 63 | 14.90% | 345 | 21.70% |
| Nausea | 19 | 11.20% | 70 | 16.60% | 292 | 18.40% |
| Coughing | 30 | 17.70% | 47 | 11.10% | 264 | 16.60% |
| Weakness | 19 | 11.20% | 49 | 11.60% | 251 | 15.80% |
| Malaise | 16 | 9.40% | 41 | 9.70% | 243 | 15.30% |
| Dyspnea | 16 | 9.40% | 34 | 8.00% | 206 | 13.00% |
| Shock | 12 | 7.10% | 30 | 7.10% | 194 | 12.20% |
| Stomach diseases | 12 | 7.10% | 30 | 7.10% | 154 | 9.70% |
| Swelling | 18 | 10.60% | 30 | 7.10% | 146 | 9.20% |
| Fear | 5 | 2.90% | 29 | 6.90% | 148 | 9.30% |
| Spots on skin | 14 | 8.20% | 53 | 12.50% | 134 | 8.40% |
| Pneumonia | 20 | 11.80% | 24 | 5.70% | 149 | 9.40% |
| Exanthema | 15 | 8.80% | 96 | 22.70% | 97 | 6.10% |
| Common cold | 11 | 6.50% | 20 | 4.70% | 132 | 8.30% |
| Seizures | 9 | 5.30% | 26 | 6.20% | 131 | 8.20% |
| Pleural diseases | 11 | 6.50% | 41 | 9.70% | 133 | 8.40% |
| Sore to touch | 8 | 4.70% | 36 | 8.50% | 114 | 7.20% |
| Alopecia | 5 | 2.90% | 28 | 6.60% | 115 | 7.20% |
| Stress | 5 | 2.90% | 20 | 4.70% | 117 | 7.40% |
| Diarrhea | 9 | 5.30% | 75 | 17.70% | 77 | 4.90% |
| Constipation | 7 | 4.10% | 28 | 6.60% | 101 | 6.40% |
| Headache | 10 | 5.90% | 25 | 5.90% | 101 | 6.40% |
| Exhaustion | 6 | 3.50% | 18 | 4.30% | 101 | 6.40% |

Please note that these symptoms were observed among patients with NSCLC and their caregivers who are using publicly available health-related social media and may not be representative of the whole NSCLC population.
